# A Case of Two-stage Surgery Using Intracranial Pressure Monitoring for Hemorrhagic Infarction during Direct Oral Anticoagulant Medication

**DOI:** 10.31662/jmaj.2023-0087

**Published:** 2023-09-20

**Authors:** Tatsuya Tanaka, Xuan Liu, Nobuaki Momozaki, Eiichiro Honda, Eiichi Suehiro, Akira Matsuno

**Affiliations:** 1 Department of Neurosurgery, International University of Health and Welfare, School of Medicine, Narita Hospital, Narita, Japan; 2 Department of Neurosurgery, Shiroishi Kyoritsu Hospital, Shiroishi, Japan; 3 Department of Neurosurgery, Imari Arita Kyoritsu Hospital, Arita, Japan

**Keywords:** intracranial pressure monitoring, hemorrhagic infarction, direct oral anticoagulant, decompressive craniectomy

## Abstract

Direct oral anticoagulants (DOACs) are considered to cause a few hemorrhagic complications, including hemorrhagic infarction; these are administered in the acute phase of cerebral infarction for secondary prevention of cerebral embolism. Hemorrhagic infarction with cerebral herniation requires urgent decompressive craniectomy and can become fatal. Perioperative management is challenging because patients are often on antithrombotic therapy. In this study, we report on a case of a 61-year-old man with left-sided hemiparesis and impaired consciousness; he suffered from a hemorrhagic infarction with cerebral herniation during oral DOAC treatment after endovascular recanalization for the middle cerebral artery occlusion. As the patient was on apixaban for <3 h, performing decompressive craniectomy was considered difficult to stop hemostasis. We then opted to perform a small craniotomy to remove the hematoma, control the intracranial pressure (ICP), and administer fresh frozen plasma. We waited for the effect of apixaban to diminish before performing decompressive craniectomy. Gradually, his level of consciousness was noted to improve. Hemorrhagic cerebral infarction while on DOAC medications can be safely treated with small craniotomy and ICP monitoring followed by decompressive craniectomy. Thus, this case highlights the value of staged surgery under ICP monitoring in the absence of an immediate administration of DOAC antagonists.

## Introduction

The incidence of symptomatic hemorrhagic infarction or intracranial hemorrhage in patients taking early direct oral anticoagulants (DOACs) within 90 days after stroke onset has been reported to be 0.56%-0.90% ^(1), (2)^. Hemorrhagic cerebral infarction can sometimes become fatal. Thus, urgent decompressive craniectomy is required in cases of hemorrhagic infarction with cerebral herniation to save lives. However, perioperative management is challenging because patients are often on antithrombotic therapy.

In severe traumatic brain injury, stepwise intracranial pressure (ICP) control under ICP monitoring has been recommended, following a previously reported staged surgical treatment strategy for severe head traumas ^(3), (4), (5), (6)^.

In this study, we report on a case of hemorrhagic infarction with cerebral herniation during DOAC administration, in which small craniotomy was performed to control ICP, followed by a staged decompressive craniectomy using ICP monitoring, with a good prognosis.

## Case Report

**Figure 1.**
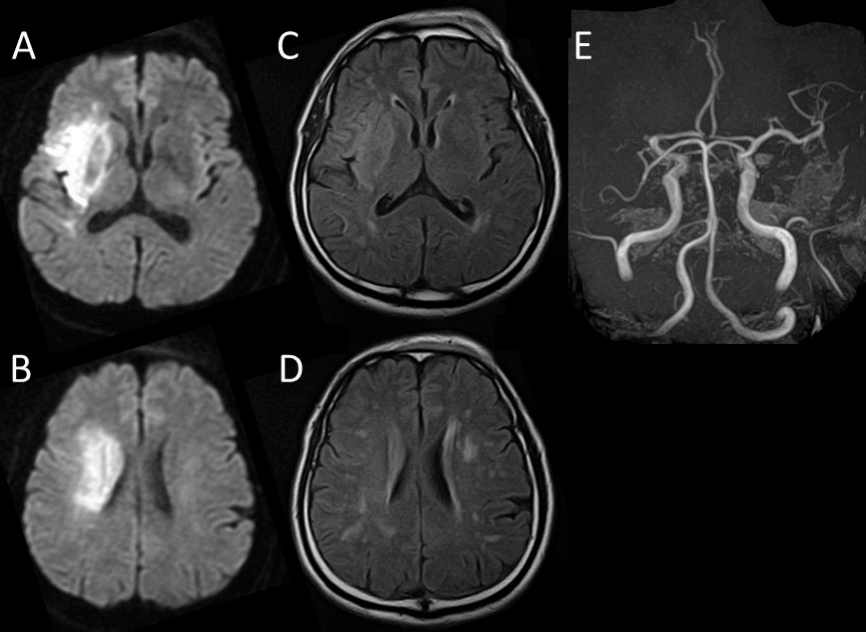
Initial magnetic resonance imaging A, B: diffusion-weighted imaging, C, D: fluid-attenuated inversion recovery and magnetic resonance angiography (E) acute infarction in the right middle cerebral artery (MCA) territory and MCA occlusion.

A 61-year-old man was transferred to our hospital via ambulance for evaluation and treatment of left-sided hemiparesis and impaired consciousness. On presentation, the cranial magnetic resonance imaging (MRI)/magnetic resonance angiography scan indicated a right middle cerebral artery (MCA) occlusion and acute and subacute infarctions in the right MCA territory (Figure 1). Moreover, atrial fibrillation was detected on electrocardiogram. Thus, the patient was directly transferred for endovascular recanalization without administering a tissue plasminogen activator. After the procedure, his status of consciousness improved, but paralysis persisted.

**Figure 2.**
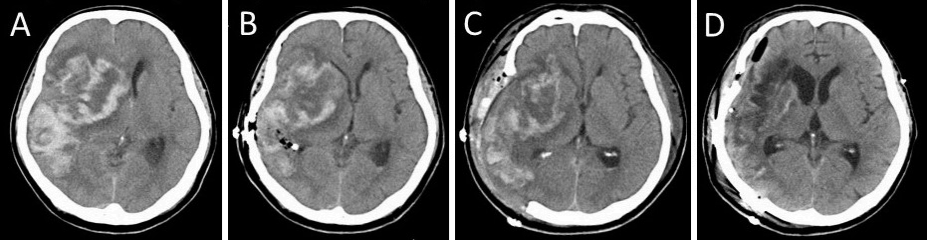
Head computed tomography (CT) indicated hemorrhagic infarction within a known cerebral infarct, midline shift, and cerebral herniation (A). Postoperative head CT indicated a progressively less midline shift after small craniotomy (B), right frontotemporal large decompressive craniectomy (C), and cranioplasty (D).

On day 9 after endovascular recanalization, treatment with 5 mg apixaban as an oral drug was initiated. On day 17 at 10.14 a.m., the patient complained of headache and lost consciousness; then, his right pupil became dilated. Head computed tomography (CT) indicated hemorrhagic infarction, midline shift, and cerebral herniation (Figure 2A).

**Figure 3.**
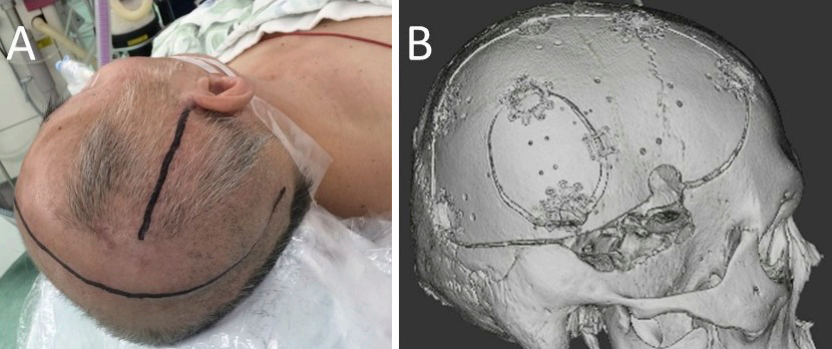
Photograph shows skin incision lines during small craniotomy and large decompressive craniectomy (A). Three-dimensional computed tomography image after cranioplasty (B).

As less than 3 h had passed after the patient had taken apixaban, it was difficult to stop the hemostasis with decompressive craniectomy. Given that the hemorrhagic infarction contains a mass of cerebral hemorrhage, its removal can temporarily reduce ICP. Thus, we have opted to perform a small craniotomy to remove the massive hematoma in the right temporal lobe, control ICP, and administer fresh frozen plasma (FFP). Decompressive craniectomy would then be performed after the effect of apixaban had diminished. At 11.25 a.m., the surgery was performed under general anesthesia. A small 5 × 4 cm craniotomy was performed (Figure 3); after rapid removal of the hematoma, a bipolar coagulation of the bleeding vessel was performed under a microscope. FFP was then administered at 0.22 p.m. An ICP monitoring system (ICP Express; Codman, Raynham) and drainage tube were placed in the hematoma cavity for ICP/cerebral perfusion pressure (CPP) monitoring. Postoperative head CT scan revealed a mild midline shift (Figure 2B). ICP dropped to <10 mmHg immediately after the surgery and then increased gradually and exceeded 25 mmHg at 3.30 p.m. Subsequently, decompressive craniectomy was performed at 5.04 p.m. The second surgery was conducted 9 h after apixaban administration. Moreover, a right frontotemporal craniectomy was performed (Figure 2C and 3).

The patient was thereafter sedated, given an infusion of concentrated glycerol/fructose solution, and hyperventilated under ventilator control to regulate ICP below 25 mmHg. Gradually, his consciousness improved. On day 62, cranioplasty was performed (Figure 2D and 3B). On day 87, the patient was transferred for rehabilitation with a modified Rankin scale of 4.

## Discussion

In this present case, the patient started taking oral DOACs on day 9 after thrombectomy for the occlusion of MCA; he then developed hemorrhagic infarction with cerebral herniation on day 17. In a recent study involving 1,797 patients who started taking DOACs after stroke onset, the incidence of symptomatic hemorrhagic infarction or intracranial hemorrhage within 90 days was 0.56%^(7)^. Early initiation of DOAC seemed suitable to reduce the risk of recurrent stroke or systemic embolism without causing major bleeding incidents ^(1), (2)^. Acute reperfusion treatment did not increase the risk of early recurrence and major bleeding in patients suffering from an atrial fibrillation-related acute ischemic stroke, who were initiated on oral anticoagulant ^(8)^. This patient had a rare symptomatic hemorrhagic infarction, although the decision to start an early DOAC medication for severe cerebral infarction was deemed reasonable.

Urgent decompressive craniotomy is commonly required for hemorrhagic infarction with cerebral herniation, but the patient was on DOAC; thus, maintaining hemostasis would have been difficult. An interval of at least 48 h after administration of apixaban is recommended for standby surgery, since it reaches maximum plasma concentration 3-4 h after administration, with an elimination half-life of 12 h ^(7)^. Active bleeding during anticoagulant therapy can be treated either by drug withdrawal, lowering systolic blood pressure below 140 mmHg, absorption inhibition by oral activated charcoal, or accelerated diuretic emptying via infusion loading ^(7), (8), (9), (10)^. Andexanet alfa, which is is a factor Xa inhibitor, has been known to neutralize the anticoagulant effect of factor Xa inhibitors, and the neutralizing effect can be maintained by continuous infusion after bolus administration, thus adjusting the time of the neutralizing effect ^(4)^. However, andexanet alfa was not available at the time. Recent guidelines have reluctantly recommended the use of high-dose prothrombin complex concentrate (50 IU/kg) for patients receiving edoxaban and apixaban when andexanet alfa is not available, but this is not indicated in Japan.

In this present case, a small craniotomy was promptly performed to control ICP while simultaneously preparing FFP administration to correct the coagulopathy caused by DOAC medication. Compared with endoscopic surgeries, small craniotomies can remove hematomas regardless of their hardness and enable the use of more hemostatic devices. A decompressive craniectomy was then performed with ICP monitoring while waiting for the effect of DOAC to diminish. Kiyohira et al. reported that performing a craniotomy for acute subdural hematoma following rapid control of ICP and correction of coagulopathy by perforation, followed by a standby craniectomy, has resulted in a lower intraoperative blood transfusion volume and a better prognosis rate than craniotomy performed without correction of coagulopathy ^(4)^. The benefits of ICP/CPP have been reported in cases of severe head trauma, and ICP/CPP monitoring has been linked to reduced mortality, with recommended levels of CPP maintained between 50 mmHg and 70 mmHg and ICP between 15 mmHg and 25 mmHg ^(3), (6)^. ICP monitoring allows time for coagulation to normalize; this enables the surgeon to perform surgery before the recurrence of cerebral herniation.

In this current scenario, coagulation abnormalities cannot be monitored when the patient is on DOACs. In the event of bleeding, administration of antagonists in addition to general measures such as blood pressure control is required. The results obtained in the management of our patient suggest the value of small craniotomy followed by ICP monitoring in patients with DOAC, especially in situations where antagonists cannot be immediately administered. ICP monitoring determine if and when additional surgery will be performed. Decompressive craniectomy can be performed safely using this method, which may contribute to improved outcomes.

## Article Information

### Conflicts of Interest

None

### Author Contributions

Tatsuya Tanaka took care of the patient and was involved in the writing, design, and editing of the manuscript. Xuan Liu, Nobuaki Momozaki, and Eiichiro Honda took care of patient and were involved in the editing of the manuscript. Eiichi Suehiro and Akira Matsuno were involved in the editing of the manuscript.

### Informed Consent

The patient had provided consent and agreed for the publication of the case in an academic journal granted his identity remain anonymous.

### Approval by Institutional Review Board (IRB)

Not applicable
